# Cell state plasticity, stem cells, EMT, and the generation of intra-tumoral heterogeneity

**DOI:** 10.1038/s41523-017-0012-z

**Published:** 2017-04-19

**Authors:** Geoffrey M. Wahl, Benjamin T. Spike

**Affiliations:** 1 The Salk Institute for Biological Studies, 10010 N. Torrey Pines Road, La Jolla, CA 92037 USA; 2 Huntsman Cancer Institute, The University of Utah, 2000 Circle of Hope, Salt Lake City, UT 84112 USA

## Abstract

Cellular heterogeneity in cancer represents a significant challenge. In order to develop effective and lasting therapies, it is essential to understand the source of this heterogeneity, and its role in tumor progression and therapy resistance. Here, we consider not only genetic and epigenetic mechanisms, but also inflammation and cell state reprogramming in creating tumor heterogeneity. We discuss similarities between normal mammary epithelial developmental states and various breast cancer molecular sub-types, and the cells that are thought to propagate them. We emphasize that while stem cell phenotypes and mesenchymal character have often been conflated, existing data suggest that the combination of intrinsic genetic and epigenetic changes, and microenvironmental influences generate multiple types of tumor propagating cells distinguishable by their positions along a continuum of epithelial to mesenchymal, stem to differentiated and embryonic to mature cell states. Consequently, in addition to the prospect of stem cell-directed tumor therapies, there is a need to understand interrelationships between stem cell, epithelial–mesenchymal, and tumor-associated reprogramming events to develop new therapies that mitigate cell state plasticity and minimize the evolution of tumor heterogeneity.

## Introduction

### Something old, something new: explaining cancers’ phenotypic heterogeneity based on what scientists from Darwin to Dvorak knew

Cancer progression is often viewed as the result of forces acting on cells in the crucible of Darwinian selection taking place within an evolving tumorous organ. In this context, the cells most likely to survive would be those ‘most responsive to change’, as they would possess the phenotypes needed to survive, proliferate, disseminate, and resist attempts at therapeutic elimination. Though Darwin actually did not originally articulate the phrases “survival of the fittest”, or “survival of those most adaptive to change” (*see* Note), these inferences from his theory of natural selection do apply to tumor progression and relate to selective pressures generated by both the epithelial and stromal tumor components. The importance of selection for “fit and adaptive” tumor cells conforms with the observation that tumor cells manifest remarkable phenotypic heterogeneity, even within a single tumor, and that subsets of cells seem particularly adept at meeting the challenges imposed by inconstant microenvironments, therapeutic interventions, and the dramatic habitat changes that accompany metastasis. In cancer, as in Darwin’s theory of natural selection, the robustness of the overall system rests on the phenotypic variation in the population and the ability of individuals or small communities to thrive in new environments. Their ability to adapt to changing microenvironments and to modify their surroundings enables cancer cells to evolve new cellular ecosystems. The inexorable variations in cellular phenotype and associated cellular adaptive potential make cancers among the most difficult diseases to treat.

Intra-tumoral heterogeneity (i.e., the phenotypic variation among cells arising from genetic, epigenetic, and environmental influences) can confound taxonomic classification and can render “precision” medicines directed against a single cellular phenotype or target ineffective. Metrics of treatment efficacy based on initial responses to surgical debulking and therapy are, therefore, unlikely to be accurate due to rare and persistent phenotypic variants that are present at diagnosis or acquired during progression. As such, intra-tumoral heterogeneity presents a key challenge to developing effective cancer treatments. Conversely, tumor cell heterogeneity may point to untapped therapeutic vulnerabilities and new, more effective routes to cancer control.

Beginning in Darwin’s own time, insights from biologists and physicians studying cancer initiation and progression have provided important clues to understanding the origins of the cellular heterogeneity evident under the microscope. Studies from the pathologist F. Durante in the mid 1800’s led him to anticipate the importance of the reciprocal relationships between tumor epithelium and stroma when he said: “Elements which have retained their […] embryonal characteristics in the adult organism, or have regained them through some chemico-physiologic deviation, represent […] the generative elements of every tumor variety and specifically those of a malignant nature. Such elements may remain enclosed within matured tissues for many years, giving no indication of their presence, until an irritation—a simple stimulus suffices—rekindles their vital cellular activities… “ (F. Durante in ref. 1). Durante’s prediction shares some similarities with the embryonic rest hypothesis put forth by Cohnheim and Virchow that formalized a theory proposing that tumorigenesis arises from cells arrested in an embryonic-like state.^1^ However, if we apply modern terminology to Durante’s proposal, we can see that he brilliantly anticipated the possibility that dormant cells could be induced to proliferate, that inflammation could be a relevant stimulus, and that an inflammatory microenvironment might enable adult, dormant cells to de-differentiate, or reprogram, into an embryonic like state. Similarly, Pierce and colleagues have referred to cancers variously as “caricatures of development” or “caricatures of tissue renewal.” Here, the word “caricature” was carefully chosen because it conveys a “gross exaggeration of a normal characteristic”.^2^ Pathologic analyses of many cancers led to the observation that certain leukemias, teratocarcinomas, germ cell tumors, and other solid tumors contain cells representing various states of differentiation, including some speculated to be “stem cells” (see refs. 2, 3). These tumors were inferred to have mutations that prevented differentiation, leading to the concept of “maturation arrest” as a mechanism of tumor progression.

One interpretation of tumor cells exhibiting different states of differentiation is that they are attempting, but failing, to re-establish tissue homeostasis. This view is compatible with Dvorak’s conceptualization of tumors as “wounds that do not heal”.^4^ He observed that tumor stroma contains the cellular constituents produced during a wound response, suggesting the failure to heal may be due as much to abnormalities in the stroma as in the cancer cells themselves. Importantly, he also noted that tumor growth can be limited by the type and amount of stroma available to support the aberrant epithelium.^5^ This view suggests a necessary evolution of the epithelium to deal with a changing stroma, and induction of metabolic and epigenetic changes in the stroma by epithelial signals to enable continued tumor growth. These interactions likely represent a caricature of the interdependence of these two compartments in normal tissues. An important implication of Dvorak’s proposal is that mutations in the tumor should affect the type of stroma present, and that the aberrant co-evolving stroma should reciprocally influence tumor cell growth. These predictions are supported by decades of independent studies (for review, see ref. 6). Conversely, normal stroma can prevent malignant cells from generating cancers.^7–9^ However, another interpretation that is also compatible with the data is that tumor cells coexisting in multiple differentiation states may support more robust tumor growth, or promote survival under environmental challenge, thereby strengthening overall tumor fitness.

A unifying observation from the above examples is that tumor cells, in meeting the evolutionary pressures created by their own genomic changes, their local aberrant micro-environments and the consequent systemic perturbations that arise, borrow extensively from the normal biological repertoire to attempt to reacquire a homeostatic balance. This suggests that (1) the variety of phenotypes a cancer cell is likely to assume are ultimately limited, (2) developmental paradigms dictate cancer phenotypes, and (3) there may be targetable intercellular communication dependencies (accessible to drugs working in the extracellular space) even in tumors driven by abnormalities in intracellular signaling pathways. However, being minimally constrained by normal tissue structure and function, cancers may elaborate extravagantly on developmental themes.

### Mechanisms for generating intra-tumoral heterogeneity and tumor cell adaptation

Genetic mechanisms contribute significantly to the ability of cancer cells to adapt to aberrant tumor microenvironments, and conversely, such microenvironments likely contribute to genome destabilization.^10–12^ The development of ever more sensitive methods for analyzing genomic rearrangements (e.g., amplifications, deletions, translocations, inversions, and point mutations) at the single cell level are revealing both the dynamics of intra-tumoral genetic landscape changes and mechanisms of tumor evolution. For example, single nucleus DNA sequencing has shown that triple negative breast cancers (TNBCs) [breast cancers lacking estrogen and progesterone receptors, and that do not overexpress human epidermal growth factor receptor 2 (Her2)] contain large-scale chromosome aberrations that arise early during tumor progression.^13–15^ This is consistent with p53 inactivation being an early or initiating event in these tumors, as loss of p53 function creates a permissive environment for genome destabilization.^16–19^ Following the chromosome instability phase, TNBCs accumulate point mutations at 10-fold higher frequencies than normal cells.^13^ This increased mutation rate further contributes to intra-tumoral heterogeneity. Rare genetic variants within these genetically complex tumors can provide sources of resistance to chemotherapeutic agents, leading to treatment failure.

Epigenetic changes provide a second mechanism for generating intra-tumoral heterogeneity.^20^ Alterations in epigenetic chromatin control pathways may be critical for tumor cells to adapt to inflammatory microenvironments, therapeutic intervention, and other challenges presented during tumor progression. Such alterations can result from mutations affecting DNA methyltransferases, histone modifying enzymes, and chromatin remodelers, which together comprise the molecular machinery that creates and interprets the histone code that contributes to gene regulatory mechanisms (for review, see ref. 21). Epigenetic control may also be affected directly, or indirectly, by mutations that disable cell cycle regulators, such as the p53 tumor suppressor.^22, 23^ Disabling p53 function can also increase the frequency at which fully differentiated cells reprogram to pluripotency,^24, 25^ a theme expanded upon below. The frequent inactivation of p53 by multiple mechanisms during cancer progression could similarly relax differentiation control through epigenetic mechanisms.^26^

Most papers on epigenetics have focused on the writers, readers and erasers that create, interpret and reverse epigenetic marks.^27^ However, more recently, importance has been drawn to the “ink” that generates epigenetic marks, such as methyl and acetyl groups.^28^ These modifications require the genesis of activated metabolic intermediates such as S-adenosylmethionine for methylation, or acetyl co-A, for acetylation.^28^ Recent data emphasize how the activity of metabolic pathways can enable cancer cells to adapt to changing environments, and how mutations in enzymes such as IDH1/2 or promiscuous activity of lactate dehydrogenase can alter histone and DNA methylation patterns, perturbations implicated in several tumor types (e.g., see ref. 29 for review). Importantly, and in contrast to genetic alterations, epigenetic contributions have the capacity to change through cell division, thereby providing opportunities for phenotypic variation in response to acute, oscillating, intracellular or micro-environmental changes that require cycles of adaptation for survival.^30^ Furthermore, aberrant epigenetic profiles need not be driven by direct mutation but may reflect metastable changes in tumor cells as they respond to altered tumor microenvironments and new tissue-specific signals during metastasis. The differences in tumor cell metabolism, which arise as the consequence of tumor specific mutations as well as responses to microenvironmental changes, can directly impact epigenetic modifications.^28^ In addition to intra-epithelial factors controlling gene expression in tumor cells, microenvironmental cues, such as Tgf, Pdgfb, Met, Egf and Fgf from tumor-associated macrophages, fibroblasts or other stroma can also lead to silencing of epithelial genes such as E-Cadherin. And, epithelial cell phenotype can be shifted to a mesenchymal state by hypoxia mediated upregulation of Twist, Snail and Zeb family transcriptional regulators and jumonji domain histone demethylases.^31–36^

Similarly, transient stress response mechanisms may underlie an additional level of tumor cell variance and adaptability. For instance, zones of nutrient or oxygen restriction in tumors may trigger intrinsic, acute adaptive responses and have been reported to contain distinct tumorigenic cell subpopulations.^37^ It is also well established that stress-induced proteins known to mediate survival of normal cells in the context of tissue disruption or exogenous stresses, including NF-kB, GRP78 and many others, correlate with poor prognosis, suggesting that stressful tumor microenvironments play critical roles in eliciting malignant cellular phenotypes.^38, 39^ As healthy tissues would generally lack such stressed environments, we might also consider that therapeutically targeting these mechanisms should be associated with a favorable therapeutic index. Adaptive responses may be latent in normal cells, but provoked by aberrant metabolic, inflammatory, and perfusion characteristics of the tumorous organ. Together, genomic rearrangements, point mutations, epigenetic variations, and biological responses to diverse stresses provide powerful mechanisms for adapting to both unstable tumor genomes and metastable microenvironments.

### Normal breast development and cell state plasticity induced by tissue architecture disruption

While normal tissues lack the genetically encoded heterogeneity of many cancers, they do provide the molecular templates for the developmental plasticity and stress responses from which mutated cancer cells derive their caricatures. In light of this, understanding how the various cell types that constitute the mammary gland arise, and examining the mechanisms of normal developmental plasticity have great relevance for understanding mechanisms by which intra-tumoral heterogeneity may arise during the evolution of breast cancer. Below, we discuss these topics with a focus on mammary gland development, though the principles likely apply to other organs as well based on the cited and other papers.

Flow cytometric separation of dissociated mouse and human adult mammary glands using a variety of markers has enabled the isolation of cellular populations enriched in basal/myoepithelial cells (e.g., EpCAMlow/med, CD49fhigh), luminal progenitors (EpCAMhigh, CD49fhigh), and the mature luminal cells (EpCAMhigh, CD49flow) that constitute the epithelial components of the mammary gland. Using these enriched, isolated cell populations, only cells in the basal/myoepithelial fraction appear to be able to function as bipotent stem cells since only they efficiently generate full, functional mammary gland outgrowths after transplantation into de-epithelialized fat pads.^40, 41^ Cells in the luminal progenitor fraction typically generate luminal-restricted colonies in vitro, and only produce mammary outgrowths at very low efficiency. Mature luminal cells lack colony-forming ability, and do not transplant. Taken together, these data indicate that mouse mammary stem cell activity in the adult is apparently concentrated in the basal/myoepithelial population.

It is important to recognize that in vitro colony formation and cell transplantation assays measure the potential of a cell to exhibit a particular activity measured under a specific set of conditions. By contrast, lineage tracing provides a method for indelibly labeling a cell to ascertain the types of descendants it generates in vivo. Several groups have employed lineage-tracing strategies, using a variety of basal-restricted or luminal-restricted marking systems to attempt to confirm the existence of, and to localize the positions of, bipotential adult mammary stem cells. Lineage tracing confirms the conclusion of in vitro studies, that the luminal compartment contains only lineage-restricted progenitors.^42–45^ However, discordant results have been obtained when tracing the fate of basal cells. While some studies support the view that a fraction of basal cells exhibits characteristics of bipotent stem cells,^46, 47^ others using some of the same Cre-driver mouse strains,^42–44, 48, 49^ or a Cre-independent stochastic gene-labeling strategy, conclude that the adult mammary gland only contains unipotent basal and luminal restricted progenitors.^50^ Another group, analyzing cell division kinetics, recently provided evidence that mammary homeostasis is maintained by a subset of proliferative, but lineage-restricted, basal, luminal, and alveolar progenitors.^51^

**Fig. 1:**
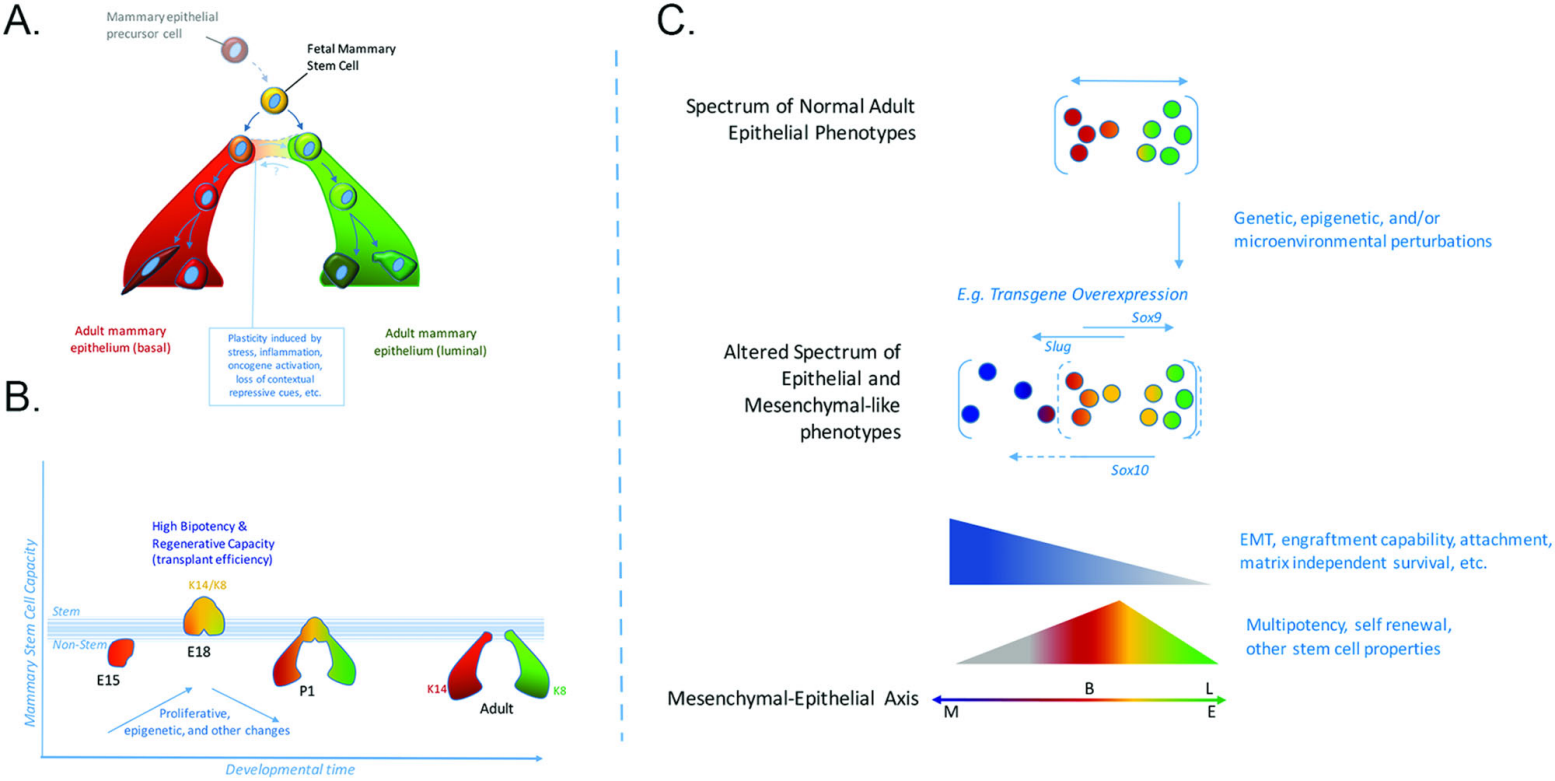
Changes in mammary stem cell activity throughout development. **a** Shown is one view of the mouse mammary cell hierarchy, starting from fetal development. Beginning at embryonic day 16 (E16), mouse cells from fetal mammary rudiments exhibit the multipotency and self-renewal functions expected for bipotential mammary stem cells. Mammary stem cell frequency increases until birth and then decreases dramatically. Currently, it is not possible to say with confidence what the frequency of adult mammary stem cells is, or whether the adult mammary gland is maintained by lineage restricted myoepithelial and luminal stem cells. By analogy with other organs, tissue disruption, inflammation and oncogene activation may enable adult mammary cells to reacquire fetal-like plasticity. This may explain the differences in stem cell frequency measured in the adult using lineage tracing, which preserves tissue structure, and transplantation, which disrupts it. **b** Probabilistic representation of mammary development depicting likelihood of cells entering a multipotent (stem) state. Changes in stem cell activity in the mammary epithelium are accompanied by cellular changes in proliferation, epigenetics, signaling, and microenvironment as well as stage specific gene expression patterns. The peak of fetal mammary stem cell activity correlates with an increased number of cells that co-express myoepithelial and luminal differentiation markers (e.g., keratins 14 and 8, respectively) and transcriptional regulators (e.g. Gata3, Elf5, Sox9, Sox10, p63). Stem cell capacity and marker co-expression are lost as development progresses. **c** Genetic, epigenetic and microenvironmental factors may expand the cellular heterogeneity of the epithelium conferring adaptability and plasticity to the population. Mesenchymal character can be conferred to epithelial cells through transcriptional regulators such as Slug and Sox 10, and may depend on both the level and duration of their expression. Stem cell activity may also be increased by appropriate balance of mesenchymal and epithelial factors (such as Slug with Sox9), or Sox10 with other factors yet to be identified, or other balanced combinations of luminal and myoepithelial specifiers. Characteristics such as EMT, multi-lineage potential and self-renewal may make certain cells better able to adapt to stresses such as those encountered in transplantation, tumor progression, migration and metastasis, or drug challenge. The position of the normal basal (B) and Luminal (L) compartments along the mesenchymal (M) to epithelial (E) axis is shown

Studies of the embryonic mammary gland have also yielded evidence for the existence of a bipotent stem/progenitor cell population.^52^ Importantly, and in contrast to the studies performed with adult tissues, in vitro sphere formation, limiting dilution transplantation,^52, 53^ and lineage tracing analyses ^42, 44^ have produced concordant results showing that fetal mammary stem cells (fMaSCs) are bipotent and become lineage restricted after birth. Interestingly, fMaSCs begin to be measureable starting at about embryonic day 16, when rudiments begin to develop exploratory sprouts that invade through the surrounding mesenchyme and enter the fat pad.^52^ The associated invasion and remodeling of cell–cell and cell–matrix interactions resembles aspects of wounding and metastasis, and expression signatures for these physiological processes are clearly evident in cell populations enriched for fMaSCs and in the surrounding stroma.^52^ One consensus model for mouse mammary development, therefore, envisions a bipotent stem/progenitor arising in mid-gestation embryogenesis, increasing in abundance until just before birth, and then decreasing significantly after birth to generate lineage-committed basal/myoepithelial and luminal progenitors. The latter then generate a subset of alveolar progenitors that fuel alveogenesis during pregnancy (Fig. 1).

We suggest that contextual differences in the biology of the embryonic and adult mammary glands can explain the abundance of bipotent stem cells in the former, and their significant decrease, or possibly absence, in the latter. We also suggest that the existing data indicate a potential for plasticity in normal mammary cells that may be evoked or enhanced by conditions existing during tumorigenesis and progression. Prior to acquisition of the stem-cell state in mid-gestation, the majority of embryonic mammary cells express the basal cytokeratin 14 but not the luminal cytokeratin 8, which is reasonable as they derive from migration of epidermal cells, and they are not proliferating.^54^ A robust proliferative program is activated at about embryonic day 15, just prior to the onset of stem cell activity.^55, 56^ Proliferation continues to increase until birth, and correlates with a progressive increase in stem cell activity. A reasonable inference is that proliferation facilitates the epigenetic reprogramming that converts keratin 14-expressing cells into a mixed phenotype population of cells that expresses both basal and luminal cytokeratins (Fig. 1).

The embryonic mammary rudiment is also undergoing profound morphologic changes as the stem cell state is acquired. The mammary rudiment is extending primitive ducts through a mesenchymal sheath into the primitive fat pad, and a lumen is starting to appear, creating for the first time a distinction between extracellular matrix-proximal and lumen-facing cells. Thus, cells in the embryonic rudiment must be able to respond to significant changes in the microenvironment, including exposure to diverse growth regulatory signals. By contrast, the adult gland has few proliferating cells, and has well demarcated and relatively static basal and luminal boundaries. The cell–cell and cell–matrix contacts that generate proliferative constraints are preserved in lineage tracing experiments, and this may explain why this method reveals more limited developmental potential. Importantly, all methods that involve cell dissociation to measure stem cell activity, such as in vitro sphere formation, or transplantation of fluorescence activated cell sorting (FACS)-fractionated cells or dissociated whole cell populations subjected to bar-coding,^57^ are likely to destroy homeostatic, anti-proliferative signals created by cell–cell and cell–matrix associations in the intact adult gland. Thus, when basal cells are freed from luminal cells, and are then exposed to the wounding environment generated in the de-epithelialized fat pad and the laminin-rich matrix (Matrigel) used for transplantation, they may acquire proliferative potential, and the epigenetic alterations and cellular reprogramming that convert basal/myoepithelial cells into bipotential progenitors. This model is consistent with observations that co-transplanting basal and luminal cells can profoundly affect mammary reconstitution efficiency,^42, 49^ and that the transplantation procedure can cause cells to acquire new fates.^58–60^ It is also possible that appropriate conditions could be found under which luminal cells could also acquire bipotency. Consistent with this idea, there is strong evidence that reprogramming can occur in vivo, and that normal tissue architecture suppresses reprogramming events required for both tissue repair and oncogenesis.^9, 61^ As one example, genetic ablation of basal airway cells in the lung enables luminal secretory cells to de-differentiate into basal stem-like cells.^61^ These reprogrammed basal-like cells proliferate in response to tissue damage, and generate the cell types required for lung repair. In this case, the normal cell environment prevents oncogenic cell reprogramming, and the injurious removal of basal cells creates a permissive environment (e.g., eliminates inhibitors) for trans/de-differentiation of luminal cells into the bipotential basal (stem) cells required for tissue repair. A normal microenvironment will also suppress somatic evolution at the genetic/epigenetic level. The maintenance of tissue microenvironments during youth has been proposed to promote stabilizing selection in stem and progenitor cell pools, thus disfavoring cells with phenotype-altering mutations (including oncogenic ones).^62^

### Cell state reprogramming associated with breast oncogenesis

Breast cancers comprise a diverse group of diseases, some of which are distinguishable by their expression of therapeutically actionable, functionally relevant proteins, such as estrogen and progesterone receptors (i.e., ER and PR), and Her2. By contrast “TNBCs” express low or undetectable levels of ER, PR, and HER2. These classifications have led to significant improvements in treatment outcomes, and have enabled the use of treatments likely to be most effective based on known tumor genetic characteristics. Breast cancers have also been classified using agnostic molecular profiling approaches that cluster tumors based on the relatedness of their gene expression signatures and similarities to normal mammary cell subpopulations.^63–65^ This has resulted in subtypes referred to as: (1) “normal-like”, which contain either a predominance of normal cells, or tumor cells that are highly related to the normal breast epithelium, (2) two luminal-related subtypes (Luminal A and B), the majority of which are ER + and comprise 60–70% of all breast cancers, (3) a Her2-expressing subtype, some of which are ER+, and (4) a complex group of TNBCs. These classifications add molecular precision to pathologic diagnoses, enable risk of recurrence probabilities to be added to the therapeutic decision tree, and have potential for identifying new therapeutic targets.^63, 66^

TNBCs exhibit substantial intra-tumoral heterogeneity, which likely underlies their propensity to develop resistance to available chemotherapies. Transcriptomic and pathologic analyses indicate that TNBCs can be divided into different subtypes.^67–70^ The “basal-like” subtype has been so designated based on the expression of keratins 5, 6 and 14—proteins typically found in the basal/myoepithelial layer of the mammary gland. By contrast, the “Claudin low” subtype of TNBC (so called because of low expression levels of claudins, which mediate cell-cell interactions through tight junctions)^71^ are strongly enriched in “basal”/mesenchymal features. These tumors are typically dominated by cells lacking E-cadherin and basal and luminal cytokeratins, and they express vimentin or smooth muscle actin.^72^ Importantly, while such designations have been used to suggest a basal cell of origin for these tumors, genes such as keratin 5/6 or 14 may also be expressed in the luminal layer,^73, 74^ perhaps influenced by microenvironmental conditions.^75^ It is also important to consider that the vast majority of human breast cancers likely arise from luminal cells.^73^ Thus, it may be more appropriate to infer that the gene expression signature of basal-like breast cancer (BLBC) reflects not necessarily the cell of origin, but the state to which the tumor has evolved at the time of diagnosis.

How might basal-like tumors be generated from a luminal cell? Studies with mouse models show that tumors resembling human BLBC can be generated by inactivating BRCA1 or by introducing mutations in the catalytic subunit of phosphatidyl inositol 3-kinase (PIK3ca) in luminal, but not basal cells.^76–78^ Furthermore, humans that carry a BRCA1 mutation are at increased risk of developing BLBC, and have been reported to have an increased number of luminal progenitor cells, based on flow cytometric analyses.^79^ These studies suggest that, in the context of the appropriate mutational background and microenvironment, differentiation state plasticity may occur. Indeed, PIK3ca mutations appear to be able to induce basal-luminal, and luminal-basal transitions, and de-differentiation into a multipotent stem-like state.^76, 77^ It is important to note, however, that only a small fraction of the tumors resulting from PIK3ca mutations are mammary adenocarcinomas, and most are subtypes rarely observed in humans. Also important is that mutation of any PI3K residues are uncommon in human BLBCs according to TCGA and METABRIC sequencing analyses.^18^ In contrast, somatic PI3K activating mutations are commonly seen in human luminal breast cancers, indicating a strong cell type selectivity, though the underlying reason for this difference remains to be elucidated. It is reasonable to infer, therefore, that introducing the right kind of PI3K pathway activating lesion into the appropriate cell type and genetic context, including but perhaps not limited to relevant mutant p53 and pRb inactivating alleles, could generate tumors that resemble human BLBC.

The rapidly growing field of induced pluripotentiality shows how cancer-relevant mutations can promote de-differentiation to generate stem-like cells. All terminally differentiated cell types analyzed thus far, ranging from fibroblasts to immune cells that have undergone V-D-J rearrangement, can be converted to a pluripotent stem-like state in vitro via the introduction of four (or fewer) genes.^80–83^ Importantly, inhibiting p53 function by diverse strategies increases reprogramming efficiency in vitro,^24, 84–86^ and mutations that compromise Rb function significantly increase reprogramming efficiency beyond that achieved by p53 inactivation alone. These findings are relevant to TNBC, as many are likely initiated by *p53* mutations, and all have high frequencies of p53 and pRb inactivation.^18^

The cellular diversity and transcriptomic characteristics of basal-like subtype of TNBC suggest that it may provide a naturally occurring example of de-differentiation facilitated by p53 and Rb dysfunction, augmented by dysregulated growth control pathways created by large scale chromosome changes and point mutations. BLBCs are referred to as “undifferentiated” due to their lack of an organized ductal structure. BLBCs contain cells that express basal cytokeratins (e.g., K14, K5), cells that express luminal cytokeratins (e.g., K8, K18, K19), and cells that express both (K14, K8/18) or neither.^87, 88^ Thus, BLBC cellular variants span the luminal-to-basal differentiation spectrum, including putative stem/bi-potential (K14/K8/K18 co-expressing) progenitors.^53, 89^ BLBC cells lacking keratin and EpCAM, and expressing vimentin or smooth muscle actin appear to have acquired mesenchymal-like characteristics that are not readily detected in the epithelium of the normal gland. Thus, BLBC appear to have cells spanning the spectrum from potential progenitors to mesenchymal-oriented variants.

Several observations suggest that cellular diversity in BLBC may derive, at least in part, from the presence of cells that resemble multipotent embryonic-like cells. First, transcriptomic analyses of bulk BLBC tumor samples reveal strong similarities to the signatures of embryonic, stem-like cells ^26, 90^ and mouse fMaSCs.^52^ Like fMaSCs, BLBCs also contain cells co-expressing basal and luminal cytokeratins. fMaSCs are also effectively ER^–^, PR^–^, and Her2 low, though their growth is augmented by signaling through EGFR family members,^52^ as can occur in BLBC.^91, 92^ Taken together, the data indicate that BLBCs contain stem-like cancer cells (SLCC) that share some properties of fMaSCs, which we will refer to as “SLCC” below. Importantly, individual tumors representing other breast cancer subtypes also exhibit different degrees of enrichment for the fMaSC signature,^52^ suggesting that many breast cancer subtypes may have SLCC. SLCC differ significantly from “cancer stem cells” (CSC) as discussed below.

SLCCs are defined by their transcriptional relatedness to functionally defined, highly enriched stem cell populations, but have not been isolated or characterized functionally. Importantly, the presence of embryonic stem cell and fMaSC signatures in BLBC correlates with poor prognosis ^52, 90^ and p53 inactivation.^26^ Recent refinement of the fMaSC signature into sub-signatures reveals that some sub-signature features (e.g., relatedness to basal cancers) predict better chemotherapy response, whereas others predict worse chemotherapy outcomes (e.g., relatedness to luminal tumors).^65^ This is consistent with the multipotentiality of this primitive cell population. If BLBCs do originate from differentiated luminal cells, or from luminal progenitors, then the cellular heterogeneity evident in BLBCs may arise, in part, from cancer cells that have de-differentiated to an embryonic-like state through the acquisition of fMaSC-like stem cell programs. Of note, the identification of fMaSC-expression signatures in bulk, heterogeneous BLBC tumor cell populations indicate that either the SLCC constitute a prominent fraction of the tumor mass, or that in aggregate, the heterogeneous tumor cell population produces a signature representative of the fMaSC cell population.

### Distinguishing cells resembling adult MaSCs and fMaSCs in breast cancer subtypes

As the vast majority of breast cancers occur during adulthood, it is reasonable to consider whether adult MaSCs (aMaSCs) that have been identified and characterized over the past decade exhibit similarities to fMaSCs. It is also important to ask whether BLBC or other breast cancer subtypes contain cells that are related to aMaSCs. The existence of stem cells in the adult mammary was suggested by classic transplantation studies,^93, 94^ and the ability of a single cell to repopulate the mammary gland was inferred from retroviral tagging strategies, indicating that a single labeled cell could generate a mammary tree as a clonal outgrowth.^95^ Later, flow-sorting with different cell surface markers afforded significant purification of cells enriched in colony forming activity in vitro and mammary repopulating unit activity in vivo.^40, 41^ As few as one cell from the aMaSC-enriched population has been reported to regenerate a fully functional gland upon transplantation.^40, 41^ Note, however, that as a pure aMaSC has never been isolated or characterized, conclusions about “aMaSCs” or “aMaSC signatures” actually refer to the aMaSC-enriched population, within which the aMaSC itself, if it exists as a discrete cellular entity (see below), represents only 1–2% of the total cell content. By contrast, fMaSCs can comprise 10–50% of the cells in the most highly enriched fetal stem-enriched populations.^52, 96, 97^

**Fig. 2:**
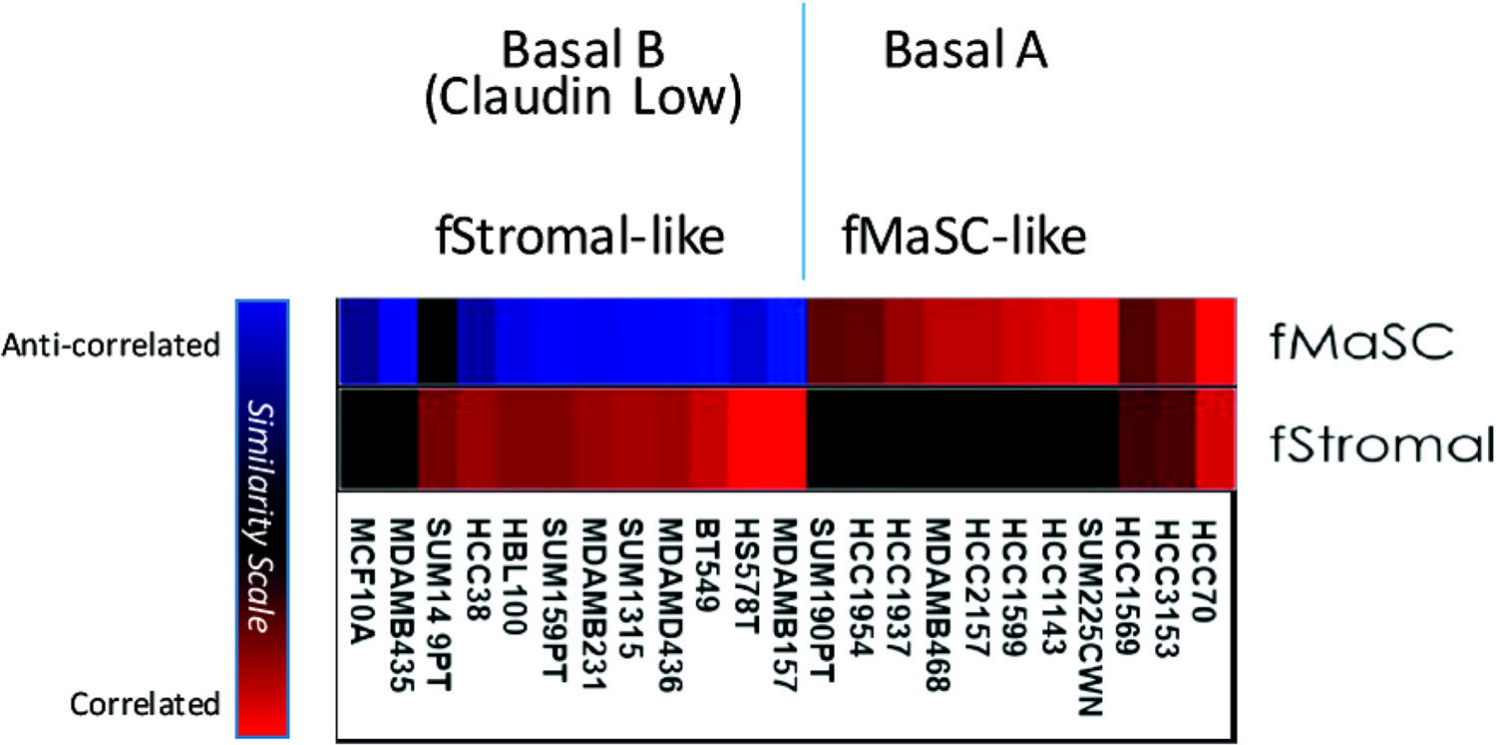
Breast cancer cell lines show differential enrichment for fetal epithelial signatures (fMaSC) and fetal non-epithelial signatures (fStromal). Basal A Cell Lines, which are most similar to ‘Basal-like’ breast cancers, show enrichment for fMaSC signatures. Basal B cell lines, which have a more mesenchymal phenotype, exhibit classic BCSC markers and correspond to ‘Claudin Low’ tumors. Claudin low tumors are enriched for non-epithelial signatures and are anti-correlated to the fMaSC state. The SUM149PT cell line shows enrichment for non-epithelial signatures similar to other cell lines classified as Basal B in the Neve et al. data set, but shows less repression of the epithelial fMASC signature.^149^ This observation, and this cell line’s subsequent characterization as Basal-like as opposed to Claudin Low, may be attributable to an intermediate or heterogeneous phenotype among its constituent cells.^150^ Claudin low cell lines also show significant enrichment for the aMaSC signature (not shown). Assessment of signature enrichment was based on signatures from Spike et al.^107^ and methods described in Segal et al.^151^

While fMaSCs and aMaSCs fulfill the classic stem cell criteria of multi-lineage generating capacity and self-renewal, they are dissimilar in many ways. First, profiling of their enriched FACS fractions has revealed significant transcriptional divergence ^52^ and their signatures are enriched in different breast cancer intrinsic subtypes. For example, aMaSC signatures are highly enriched in Claudin low tumors and Claudin low cell lines,^52^ which often contain significant numbers of classically defined breast cancer stem cells (BCSCs). Neither Claudin low tumors nor BCSC-rich cell lines show significant relatedness to fMaSCs (Fig. 2). Rather, as described above, fMaSCs are transcriptionally most related to BLBC and BLBC related cell lines (Fig. 2). One explanation for the limited transcriptional relatedness of aMaSCs and fMaSCs has to do with the purity of the populations used for ascertaining their respective signatures. Thus, the aMaSC signature, which is derived from a basal-myoepithelial enriched cell fraction,^49^ does not reveal the critical characteristics of “pure aMaSCs”, but rather of the cell mixture in which aMaSCs are at best a minority component, as suggested by recent lineage tracing studies.^48, 49^ Second, fMaSCs and aMaSCs are distinguishable using functional assays. As one example, mammosphere formation has been used as an in vitro surrogate for stem cell activity by measuring the ability of a single cell to differentiate into multiple cell types, a cardinal requirement for a stem cell. While independent analyses show that a single fMaSC has this ability,^52, 97^ the myoepithelial-enriched fraction containing aMaSCs generates mainly small spheres solely comprised of basal cells, but only a few larger multilineage spheres under conditions that generate mainly differentiated and correctly polarized spheres from fMaSCs^52, 98^ (C. Dravis and C. Trejo, unpublished results). Furthermore, in most cases the spheres generated from aMaSC-enriched populations have not been rigorously shown to be clonal, and when evaluated carefully, most arise from aggregation using the non-adherent conditions originally employed for evaluating tumorsphere formation from BCSCs.^99, 100^ Interestingly, a subset of adult cells does show multilineage differentiation potential under more permissive media conditions^98^ (C. Dravis, unpublished data). Recently, adult Lgr5-expressing basal cells were reported to form spheres containing basal and luminal cells, as long as the growth conditions included R-spondin, Wnt3a, and a laminin-rich matrix. Even these spheres appeared to lack polarity, did not contain a lumen, and evidence of clonal origin was not presented.^101^ The lineage tracing studies summarized above also indicate significant differences between the capacity of fetal and adult cells to serve as stem cells/bipotent progenitors. Thus, the transcriptional differences between aMaSCs and fMaSCs may actually reflect their significant functional differences. Taken together, we suggest that bipotential mammary stem cells may be restricted to a narrow interval of embryonic growth, and that bipotentiality in the adult is seen only under specific growth conditions.

**Fig. 3:**
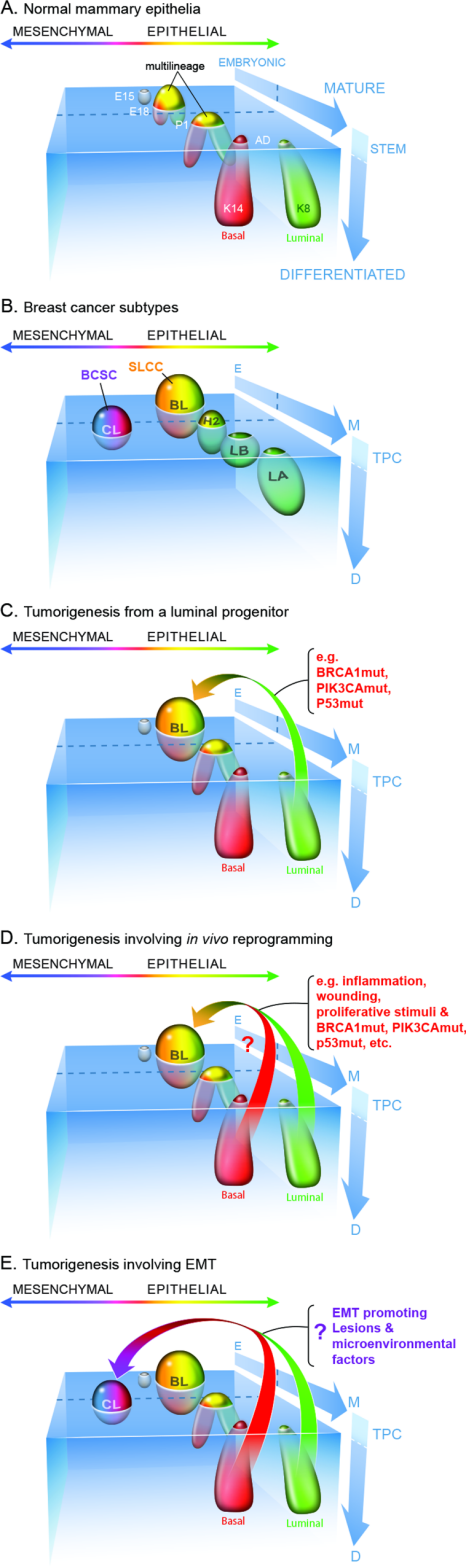
Diverse states of the normal and neoplastic mammary epithelium relative to development, stem cell differentiation, and epithelial/mesenchymal phenotypes. **a** Changes in potential for cells to enter the stem cell state throughout development can be conceptualized in a coordinate space that relates stem cell abundance and differentiation (y-axis) with developmental progression (z-axis) and epithelial/mesenchymal phenotypes (x-axis). The various cell types within the mammary epithelium occupy distinct regions in this space over the course of development (as also proposed by Granit et al.).^152^ **b** The different intrinsic subtypes of breast cancer occupy some of the same space as the normal mammary epithelium leading to partially shared gene expression profiles and operative molecular mechanisms. We suggest using tumor propagating cell (TPC) as a general term to describe cells able to generate and propagate tumor xenografts. These cells are expected to exhibit cell state plasticity to generate intra-tumoral heterogeneity in either immune compromised mice when assaying human tumor cells, or immune intact mice when using isogenic mouse cells for analysis. It remains to be determined whether TPC initiate or propagate tumors in humans. TPC may either be mesenchymally oriented as are classic “cancer stem cells” such as BCSC, or they may resemble the more SLCC that share similarities with fMaSCs. *CL* Claudin Low, *BL* basal like, *H2* Her2-like, *LB* luminal B, *LA* Luminal A. Inter-tumoral and intra-tumoral heterogeneity may further rest on local differences in expression of key regulators (e.g., Sox10, Cripto) – the expression and activity of which can be affected by autocrine or paracrine factors produced in the epithelium or microenvironment. Furthermore, genetic background and microenvironmental influences including immune responses and local and systemic stresses may impact aggressiveness by shifting the thresholds that distinguish stem cells from non-stem cells. **c** a model involving the generation of a tumor from a luminal progenitor. The Luminal progenitor already harbors some stem cell characteristics, such as cell state plasticity and a higher proliferative index that may facilitate epigenetic reprogramming to bipotentiality. Luminal progenitors appear to dedifferentiate into a bipotent-like state by activation of diverse oncogenic pathways and loss of tumor suppressors previously associated with BLBCs. **d** More mature cells may also have tumorigenic potential following tissue disruption, inflammation or other proliferative stimuli that induce facultative stem-like phenotypes. If these influences promote plasticity and reprogramming in situ, the resulting stem cell-like tumors could in fact derive from a variety of precursor cells. **e** The cell or cells of origin of Claudin low tumors remain to be defined, and this figure presents mechanisms for their genesis involving intrinsic or extrinsic EMT promoting signals. The genesis of these tumors may also depend upon induction of phenotypic plasticity and tumor cell-associated reprogramming

An extension of the above is that studies showing transcriptional relatedness between BCSC and aMaSCs do not prove that BCSCs are actually stem-like in a developmental sense, since there is no direct and consistent evidence that aMaSCs, or more specifically their reported expression signatures, represent bona fide bipotent stem cells. Indeed, a recent study analyzed the possible existence of aMaSCs during puberty, when one would expect substantial mammary cell proliferation, and when bipotential cells might be required most to fulfill the demands for extensive branching morphogenesis and multilineage tissue generation. However, what was discovered was that a group of transcriptionally heterogeneous cells in the terminal end buds, rather than a single transcriptionally defined cell type, mediate the genesis of the complex epithelial network that comprises the pubertal mammary gland.^102^ Two important inferences can be drawn from this study; (1) the stem cell state may not be generated by a unique transcriptional pattern, and (2) cells able to fulfill the multipotential lineage criterion may only be identifiable using functional assays. As pointed out elsewhere in this review, however, functional assays involving transplantation or in vitro culture only measure cellular potential under those specific assay conditions, and do not prove similar cell function in vivo. The fact that BCSC show relatively little transcriptional relatedness to fMaSCs—cells which do fulfill all of the functional criteria for bipotential mammary stem cells in vitro and in vivo—further suggests that BCSC should not be considered truly “stem cell-like” on the basis of their predominant transcriptional features. Rather, these features may phenocopy a mesenchymal-like cell state that can be achieved by cells of diverse differentiation states in response to genetic, epigenetic, or microenvironmental changes. In light of this, below we provide a new perspective in which BCSCs and SLCCs represent different cell states in a mammary lineage continuum. We suggest that these states can be generated, or altered, by conditions such as wounding or inflammation that in other systems produce cell state plasticity and likely contribute to tumor heterogeneity (Fig. 3).

### Stemness and epithelial-mesenchymal transition (EMT): different states on a differentiation continuum

Many studies over the past decade have equated stemness with the EMT, and EMT with BCSC (e.g., see ref. 103–105). It is worthwhile re-examining the seminal experiments that led to these conclusions to assess whether other interpretations should now be considered.

The now classic studies of Wicha, Clarke and colleagues showed that some breast cancers contain a subset of cells, BCSCs, that are particularly effective at generating tumors in a xenograft setting.^106^ While the original BCSCs were isolated as CD44^high^CD24^low^, it is now clear that BCSCs can be identified using a variety of markers ^107, 108^ and that multiple populations within the same tumor can contain cells exhibiting cancer stem cell (CSC) activity.^109^ This may in part be explained by technical differences,^110^ but it is also possible that the CSC phenotype may be elicited by different genetic programs, all converging on a common phenotypic state, and/or that the CSC state is itself highly plastic.^107, 111^

As the “BCSC-state” is reversible, identifying the pathways involved in entry into, and exit from, this state is a priority. In one approach, Weinberg and colleagues started with immortalized human mammary epithelial cells (MECs) derived from a reduction mammoplasty.^104^ They transduced these cells with various factors to see which could increase the number of CD44^high^CD24^low^ cells in the parental population. Snail and Twist, transcriptional regulators known to induce EMT, increased the number of CD44^high^CD24^low^ cells, which correlated with increased efficiency of mammosphere formation in non-adherent culture, and secondary mammosphere formation upon dissociation and replating. The gene expression profiles of the parental cells and the CD44^high^CD24^low^ Snail/Twist-induced variants were consistent with the epithelial parental population having undergone a mesenchymal transition. Interestingly, transplantation of the immortalized CD44^high^CD24^low^ cells, but not populations depleted of these cells, regenerated apparently full mammary glands, but no tumors. The functional characteristics of the CD44^high^CD24^low^ HMLE cells (human MECs), their similarity to BCSC, and the relatedness of BCSC to aMaSCs based on transcriptional relatedness (see above, and ref. 104) led to the proposal that a mesenchymal phenotype or EMT is a core property common to BCSC and MaSCs, and by extension, contributes to the genesis of both.^104^ While these studies were done using an immortalization procedure that compromises p53, pRb, and PI3K control, all of which can influence the probability of cell reprogramming to a stem-like state (see above), subsequent studies by Guo et al. came to similar conclusions using normal MECs.^103^ However, Guo’s studies found that instead of Snail and Twist inducing EMT and “stemness”, Slug, another EMT transcription factor, and Sox9, a transcriptional regulator involved in cell fate transitions in multiple systems,^112^ were required. The differences between these studies demonstrate that “EMT” can be generated by different factors depending on genetic background, differentiation state, and microenvironment.^105^

The data presented above beg the question: “Does EMT equal stemness?” Though elegant in concept and execution, these studies, as well as others not discussed, are open to alternative interpretations and may be influenced by assay-specific effects. For example, the mammospheres formed by the CD44^high^CD24^low^ cells were not polarized, lacked lumens, and in some cases contained a minority of keratin expressing epithelial cells. The lack of polarization is not expected for a mammosphere generated from a bona fide mammary stem cell, such as an fMaSC.^52^ Noteworthy, however, was the presence of a small minority of individual cells expressing both myoepithelial (K14) and luminal (K8/18) keratins, suggesting that some cells in the disorganized spheres may have partially phenocopied a bipotential stem/progenitor cell. Importantly, while some studies used methyl cellulose or other conditions to limit the possibility that spheres arose from cellular aggregation, there was no direct evidence that the majority of spheres arose by clonal expansion from a single founding “stem cell.” Indeed, the very low frequency at which spheres were generated from highly enriched CD44^high^CD24^low^ populations indicates that either very rare cells from this population are “stem cells”, or that most spheres arose from aggregation, as would be expected for the highly motile, aggregation-prone cells generated by EMT. It was also not demonstrated that the glandular outgrowths arose from a single initiating cell, as required by the stem cell model. Thus, the increased probability of gland formation from EMT’d mammary cells may be explained by multiple cells, each in a somewhat different differentiation state, contributing to the genesis of the observed structures. The EMT’d cells may be more effective at invading the fat pad, acquiring a blood supply, and recruiting the other progenitors needed to form a ductal network, for instance. Increased mammosphere formation and increased tumorigenicity may not result from the presence of stem-like cells, but rather from mesenchymal-oriented cells that are more motile, more invasive, and better able to grow under the experimental conditions employed.

### Epithelial-mesenchymal balance and the stem cell state

The transcriptomic, functional, and biologic data summarized above do not support the idea that EMT equals stemness, yet, they do suggest a potentially important relationship between the two. Recent studies concerning how differentiated cells are reprogrammed to pluripotency provide a conceptual basis for understanding the genesis of the stem cell state in the mammary gland (and other tissues), and for understanding how EMT and stemness may be related. There is now compelling evidence that the pluripotent stem cell state is achieved when factors that control specification of opposing lineages are expressed within a single cell.^113–115^ According to this model, the cardinal pluripotency-inducing factors, such as Oct4 and Sox2, do not act to impose a program that prevents differentiation, but rather, they act as specifiers, which, when expressed at appropriate levels, create a cellular state able to respond to microenvironmental cues to differentiate down defined lineages. Consistent with this “balanced lineage specifier model”, overexpression of either of these factors leads to differentiation. For example, Oct4 overexpression specifies mesoderm, while Sox2 overexpression leads to neuroectoderm differentiation (see ref. 113). As an extension of this model, we propose that the counterbalancing of luminal and mesenchymal (myoepithelial) lineage specifying programs is likely to play a central role in generating the mammary stem cell state and that some factors involved in EMT can contribute to this balance. The ability of Slug, a mesenchymal inducer, and Sox9, a transcriptional regulator associated with luminal specification in the mammary gland,^116^ to contribute to “stemness” in normal MECs is entirely consistent with this idea (Fig. 3). Thus, aMaSCs and BCSC may represent mesenchymally oriented cells that arose from an imbalance of mesenchymal (relative to luminal) specifiers. Instead of EMT equaling stemness, we infer that a population undergoing EMT is comprised of cells in heterogeneous states of differentiation, among which a small fraction may have balanced expression of the lineage specifiers, enabling them to transiently enter a stem or bipotential state. Changing this balance by overexpressing or underexpressing lineage specifiers then shifts the balance away from bipotentiality to predictable states of differentiation (Fig. 3).

Two lines of evidence support the balanced lineage specifier model for mammary stemness, and recent studies are providing insight into some of the relevant pathways. Profiling of individual cells within a highly enriched fMaSC population using different single cell RNA-PCR and sequencing methods^52^ (Spike et al. manuscript in preparation) shows that a significant number of individual cells in the fMaSC-enriched population express: (1) both luminal and myoepithelial keratins, (2) luminal differentiation regulators, including Sox9, GATA3 and Elf5, and (3) genes associated with myoepithelial/mesenchymal specification, such as Slug, Vimentin, Sox10 (see below), and smooth muscle actin (ACTA2). fMaSCs also express what have been considered classic CSC markers, such as CD44, and ALDH1a3, although in contrast to CSC, they express high levels of CD24, a luminal marker that is also inducible under hypoxic conditions, such as would be found in the embryonic mammary rudiment^52^ (Spike et al. unpublished data). Interestingly, Guo et al. ^103^ showed that Sox10 is an important downstream target of Slug and Sox9, and its knockdown prevents acquisition of “stemness” by MECs. We recently showed that Sox10 is both an excellent marker for prospective purification of fMaSCs, and is also essential for their function.^96^ Moreover, the level and duration of Sox10 expression critically impacts the resulting cellular phenotype. Thus, overexpression for short times is associated with increased efficiency of secondary sphere formation, a surrogate for stem-cell self-renewal. However, extended expression of Sox10 induces a motile, mesenchymal-like state, that is readily reversed upon reduction of Sox10 expression.^96^ Importantly, the motile cells have lost many epithelial markers, and gained numerous mesenchymal markers and significantly reduced their proliferation rate. Interestingly, inducing a mesenchymal-like state in fMaSCs by Sox10 overexpression is not associated with up-regulation of classic EMT regulators, such as Snail, Twist, Zeb1, etc.^96^ Reducing Sox10 enables reacquisition of bipotentiality, which is expected of fMaSCs. Thus, Sox10 overexpression in fMaSCs provides a valuable model for how cells can enter into a mesenchymal-like state, and revert from it to a bipotential epithelial state. Recent in vivo studies are also consistent with a model in which epithelial-mesenchymal plasticity mediates migration and subsequent colonization of distant sites.^117^

The balanced lineage specifier model predicts that stem cell state specifiers should be responsive to cues coming from cell-cell, cell-matrix, and paracrine factors to enable rapid responses to changing microenvironments, some of which comprise the “stem cell niche”. Consistent with the role of Sox10 as such a specifier, its expression is regulated by Fgf10 produced by the fetal stroma. fMaSCs express FGF receptors, and the Sox10 induced EMT-like state is prevented by FGFR inhibitors.^96^ In a second example, Cripto, a paracrine factor generated by stromal cells, signals through cell surface GRP78 on fMaSCs and MECs to promote EMT-related and receptor tyrosine kinase-related signaling, thereby increasing the number and self-renewal of K14/K8 putative bipotential stem/progenitors.^98^ The transcriptomic and functional data therefore suggest that while EMT does not equal stemness, it likely represents one state within a reversible differentiation continuum influenced by genetic, epigenetic, and microenvironmental factors. In this view, a combination of intrinsic and extrinsic factors influence the probability of a cell entering the stem cell state. We further suggest that certain mutational backgrounds may increase the probability of reprogramming in response to external stimuli including inflammation.^118, 119^ Similarly, the degree to which a stem cell assumes mesenchymal properties will impact its performance in most assays and in certain aspects of normal stem cell function or tumorigenesis and progression (Fig. 3).

We speculate that for long-lived metazoans, it may be beneficial to limit the number of multipotent, organ-specific stem cells in the adult, and instead rely on cell state reprogramming to generate facultative stem cells to repair local tissue damage. Could this same plasticity be an Achilles heel? Retaining these facultative abilities and associated proliferative potential would make extracellular controls that govern cessation of the wound or growth signals even more important in tumor suppression. Furthermore, if EMT contributes to metastasis, then such reprogramming may both fuel the formation of the seeds, and increase the probability of their germination in the fertile soil that creates the microenvironment suitable for their reversion into the proliferative stem-like state that contributes to tumor cell differentiation state heterogeneity.

### Tissue injury as a facilitator of cell state reprogramming

There are now many examples in which wounding and inflammation, alone or in the context of oncogenic mutations, induce cell-state reprogramming, as predicted by Durante almost 150 years ago (e.g., see ref. 120, 121). Pancreatic ductal ligation (PDL), which serves as a model of pancreatic wounding, causes broad degeneration of acinar cells.^122^ While lineage tracing studies show that PDL kills many acinar cells, the survivors exhibit gene expression signatures of embryonic multi-potent pancreatic progenitors.^123^ The reprogrammed cells can subsequently generate both acinar and ductal cells to facilitate repair.^122, 123^ Importantly, acinar cells are far more susceptible to transformation by mutant K-Ras than ductal or centro-acinar cells, a process enhanced by inflammation and involving the reprogramming of acinar cells into ductal cells.^124–127^ The reduced potential of ductal cells to transform appears to be related to greater sensitivity to p53-mediated responses.^128^ Reprogramming of basal cells in the skin to an embryonic-like stem/progenitor state has also been reported during formation of basal-cell carcinomas initiated by activation of the hedgehog and Wnt pathways.^129^

Colon tumorigenesis is another system in which a particular activating oncogenic mutation can have profoundly different effects on cancer initiation, progression, and cell state reprogramming depending on the cell type affected and microenvironmental perturbations imposed. Mutations that activate the Wnt pathway initiate formation of colon adenomas if introduced into the crypt base columnar cells that comprise one type of colon stem cell.^130, 131^ Invasive adenocarcinomas do not result in this case. On the other hand, introducing the same mutation into post-mitotic differentiated cells in the colon,^118^ believed to be tuft cells,^119^ has no effect, unless animals are also subjected to a treatment that induces chronic colonic inflammation. Under these conditions, the post-mitotic cells with Wnt activating mutations re-enter the cell cycle, de-differentiate to adopt features of the crypt-base columnar cells, and initiate formation of tumors that evolve into invasive colon adenocarcinomas. In Barrett’s esophagus, persistent tissue irritation leads either to the activation of residual embryonic stem cells, or the rare reprogramming of adult cells into an embryonic stem-like state.^132, 133^ P53 inactivation can also be an early event in the development of esophageal adenocarcinoma from Barrett’s esophagus,^133^ consistent with the ability of p53 inactivation to increase the rate at which cellular reprogramming to a stem-like state occurs.^134^ In addition to impacts on cell state reprogramming, inflammation can also promote selection for oncogenic mutations which are adaptive in the new inflamed microenvironment,^135, 136^ consistent with computational studies demonstrating a key role for microenvironmental change in somatic evolution.^137^

The studies described above for the colon and esophagus provide evidence for “facultative stem cells”, which are differentiated cells that respond to tissue damage by activating the stem cell program. Facultative stem cells can be the most abundant cell type in the organ, as in the pancreas, and appear poised to reprogram to an embryonic state. Although this capability is adaptive for tissue repair, it also means that these cells may be easily converted into cells-of-origin for cancer. This perspective reconciles two extreme models of cancer initiation, one of which posits initiation by rare stem cells, and the other by abundant, proliferative progenitors or differentiated cells. Current data suggest that elements of both models are correct. It appears that cancer-relevant mutations, when put in the context of tissue injury or chronic inflammation, can enable differentiated cells to reprogram into intermediate, stem-like states conducive to tumor growth.

### Concluding remarks: heterogeneity as a potential Achilles heel in cancer

The above summary shows why cancers have been referred to as “caricatures of normal development”,^138^ “caricatures of normal tissue renewal”,^2^ and “wounds that do not heal”.^4^ In non-pathologic situations, proper control of inflammatory responses and wound healing requires cellular interactions and the arrest of these processes when homeostasis and repair have been achieved. Mutations and other abnormalities that underlie tumorigenesis, including those that enable the genesis of an abnormal stroma, likely contribute to disease progression by preventing normal repair from occurring and by generating a persistent wound environment. Nevertheless, it is interesting to speculate that tumor heterogeneity arises, in part, because there is selective pressure to conserve some elements of normal organ structure and function, while at the same time allowing for unscheduled growth and proliferation under conditions of genomic, metabolic, nutrient, and oxygen stress. In this context, understanding whether cancers consist of communities of “cooperating or competing cellular societies” (terminology from Heppner),^139^ and the mechanisms by which these societies arise may enable the development of novel treatment strategies with high therapeutic indices.

Progress in this area is being made rapidly, as demonstrated by the publication of data confirming many of the inferences made above during the editorial review of this article. For example, several recent studies show that the challenging conditions within which tumors grow increase the probability of cell state reprogramming, and the genesis of either multipotential stem-like cells, or cells resembling another lineage that contributes to both metastasis and drug resistance. Thus, mice containing an inducible cassette able to express all four Yamanaka reprogramming factors (Oct4, Sox2, KLF4 and cMyc) can induce teratomas in many tissues, but the frequency at which this occurs is significantly increased by both p53 tumor suppressor inactivation and exposure to a senescence-like environment.^140^ It is tempting to speculate that cell reprogramming enabled by both relevant mutations and exposure to senescent cells that accumulate with age provides one explanation for the link between cancer and aging. In a second example, loss of Rb function in mouse prostate adenocarcinomas that were initiated by PTEN loss increased lineage plasticity, while additional loss of p53 enabled conversion to a neuroendocrine variant resistant to antiandrogen therapy. The lineage plasticity in this system required increased expression of the reprogramming factor Sox2 and the histone methyltransferase EZH2, and the process could be antagonized by Sox2 and EZH2 inhibitors.^141, 142^

Additional work is needed to decipher the contributions of individual clones within heterogeneous tumor societies. For example, if two clones within a cancer depend on each other for paracrine survival factors, eliminating one will likely reduce the viability of the other. By contrast, if two clones compete for survival factors, eliminating one may lead to the expansion of the other and tumor progression. Studies as far back as 1950 showed that mixing tumor subpopulations can affect tumor growth rate,^143^ immunogenicity,^144^ drug response,^145^ and metastasis.^146^ More recently, interdependent clones have been described in mammary cancers.^147^ And, in a model of metastatic lung cancer, Berns and colleagues showed that the primary tumor contained neuroendocrine and mesenchymal cells that arose from a common progenitor. Each cell type was tumorigenic, but neither was metastatic. Mixing them together, however, elicited a paracrine signal from the mesenchymal cells that caused metastatic spread of the neuroendocrine cells.^148^ Understanding when tumor clones cooperate to induce tumor cell motility and escape, and understanding similar communication between tumor clones and stroma, could lead to new approaches to mitigating disease progression, including metastasis.

As stem-like cells have now been observed in diverse cancers and are generated under conditions that lead to cancer progression,^118, 119, 129^ it will be important to address a number of outstanding questions: (1) what mechanisms mediate entry into, and exit from, the stem cell state? (2) What intercellular communication mechanisms mediate these state changes? (3) How are these mechanisms perturbed by oncogenic mutations and environmental stimuli? (4) Are the mesenchymal-oriented BCSC, or the fMaSC-related SLCC, the engines of tumor heterogeneity, or do they both simply represent alternative, interconvertible, cell states? (5) Finally, will understanding the factors that lead to these states provide new targets for more selective and effective therapies? As one example, if reprogramming to an embryonic-like state contributes significantly to progression of BLBCs, could we identify embryonic antigens selectively expressed within the tumor to use as targets for immune therapeutic approaches? Will inhibition of reprogramming factors, or epigenetic modifiers, prove to be effective and have acceptable therapeutic indices in cancers in which cellular reprogramming may be actively generating more fit, heterogeneous cancer cell societies? As cancer cells thrive in challenging and changing tumor microenvironments, achieving ‘adaptability’ and ‘fitness’ through mechanisms of cellular plasticity and reversion to an embryonic state, we should endeavor to understand these novel mechanisms and convert this formidable strength to a targetable liability.

## Note

Despite popular belief, Darwin was not the first to use “survival of the fittest” as a shorthand way to explain to the general population what he meant by “natural selection”. Rather, the British philosopher Herbert Spencer, having studied Darwin’s *On the Origin of Species* (1859) used this phrase in his 1864 book *Principles of Biology* to draw parallels between species competition in the biological world and his economic theories concerning financial dynamics. Darwin later used Spencer’s phrase in the fifth edition of *On the Origin of Species* (see B. Ratner, www.GenIQModel.com). Darwin also did not say “It is not the strongest of the species that survive, nor the most intelligent, but the ones most responsive to change.” Rather, Nick Matzke, a biology graduate student, traced that perceptive insight to a longer statement made in a 1963 speech given by Louisiana State University business professor Leon Megginson (https://pandasthumb.org/archives/2009/09/survival-of-the-1.html).
